# Abdominal pregnancy secondary to uterine horn pregnancy: a case report

**DOI:** 10.1186/s12884-023-05704-4

**Published:** 2023-06-03

**Authors:** Xingju Zheng, Yao Zhou, Zhucheng Sun, Ting Yan, Yan Yang, Rongpin Wang

**Affiliations:** 1 Department of Radiology, Guizhou Provincial People’s Hospital, Guiyang, 550002 China; 2 Department of Vascular Surgery, Guizhou Provincial People’s Hospital, Guiyang, 550002 China; 3 Department of Gynecology, Guizhou Provincial People’s Hospital, Guiyang, 550002 China

**Keywords:** Ectopic pregnancy, Uterine horn pregnancy, Abdominal pregnancy, Uterine malformation, Case report

## Abstract

**Background:**

Pregnancy begins with a fertilized ovum that normally attaches to the uterine endometrium. However, an ectopic pregnancy can occur when a fertilized egg implants and grows outside the uterine cavity. Tubal ectopic pregnancy is the most common type (over 95%), with ovarian, abdominal, cervical, broad ligament, and uterine cornual pregnancy being less common. As more cases of ectopic pregnancy are diagnosed and treated in the early stages, the survival rate and fertility retention significantly improve. However, complications of abdominal pregnancy can sometimes be life-threatening and have severe consequences.

**Case presentation:**

We present a case of intraperitoneal ectopic pregnancy with fetal survival. Ultrasound and magnetic resonance imaging showed a right cornual pregnancy with a secondary abdominal pregnancy. In September 2021, we performed an emergency laparotomy, along with additional procedures such as transurethral ureteroscopy, double J-stent placement, abdominal fetal removal, placentectomy, repair of the right uterine horn, and pelvic adhesiolysis, in the 29th week of pregnancy. During laparotomy, we diagnosed abdominal pregnancy secondary to a rudimentary uterine horn. The mother and her baby were discharged eight days and 41 days, respectively, after surgery.

**Conclusions:**

Abdominal pregnancy is a rare condition. The variable nature of ectopic pregnancy can cause delays in timely diagnosis, resulting in increased morbidity and mortality, especially in areas with inadequate medical and social services. A high index of suspicion, coupled with appropriate imaging studies, can help facilitate its diagnosis in any suspected case.

## Background

Ectopic pregnancy refers to any pregnancy in which the developing products of conception implant outside the endometrial cavity. Although the actual prevalence is unknown and may be higher because of the lack of standardized reporting systems and available outpatient treatment options, approximately 2% of pregnancies are ectopic [1]. Ectopic pregnancies are the leading cause of first-trimester mortality and are responsible for approximately 4% of all pregnancy-related mortalities [2, 3].

Abdominal pregnancy, which is an embryo or fetus in the abdominal cavity, is extremely rare, and is divided into primary and secondary types. In primary abdominal pregnancy, the embryo embeds in the normal fallopian tubes and ovaries, without uteroplacental fistula, and attaches completely to the peritoneal surface in the early stages of pregnancy [4, 5]. Secondary abdominal pregnancy often occurs after abortion or rupture of the fallopian tube pregnancy [6], and occasionally, it is secondary to ovarian or uterine pregnancy with uterine defects (such as a uterine scar, fissure, or peritoneal fistula). When the embryo falls into the abdominal cavity, part of the villus tissue remains attached to the original implantation site and grows outwards, attached to the pelvic peritoneum and adjacent organ surfaces. Abdominal pregnancy is often accompanied by abnormal placentation and insufficient blood supply, which compromises the ability of the fetus to survive to full term.

## Case presentation

A 22-year-old woman, G3P1, was admitted in August 2021 after being referred from a local hospital for amenorrhea of 23 weeks and 5 days duration.

Before referral, an ultrasound scan in the seventh week of her pregnancy showed she had a congenital uterine malformation suspicious of a bicornuate uterus. The scan also revealed an intrauterine pregnancy in one cavity, a live embryo, a small amount of fluid around the pregnancy sac, and what appeared to be some fluid in the other uterus. Her previous pregnancy, five years earlier, culminated in an emergency cesarean section for intrauterine fetal distress. She claimed to have a bicornuate uterus.

After admission, an ultrasound scan showed a live fetus in the abdominal cavity with the placenta on the right side of the abdominal cavity instead of in the endometrium. We diagnosed the woman with a case of abdominal pregnancy.

**Fig. 1:**
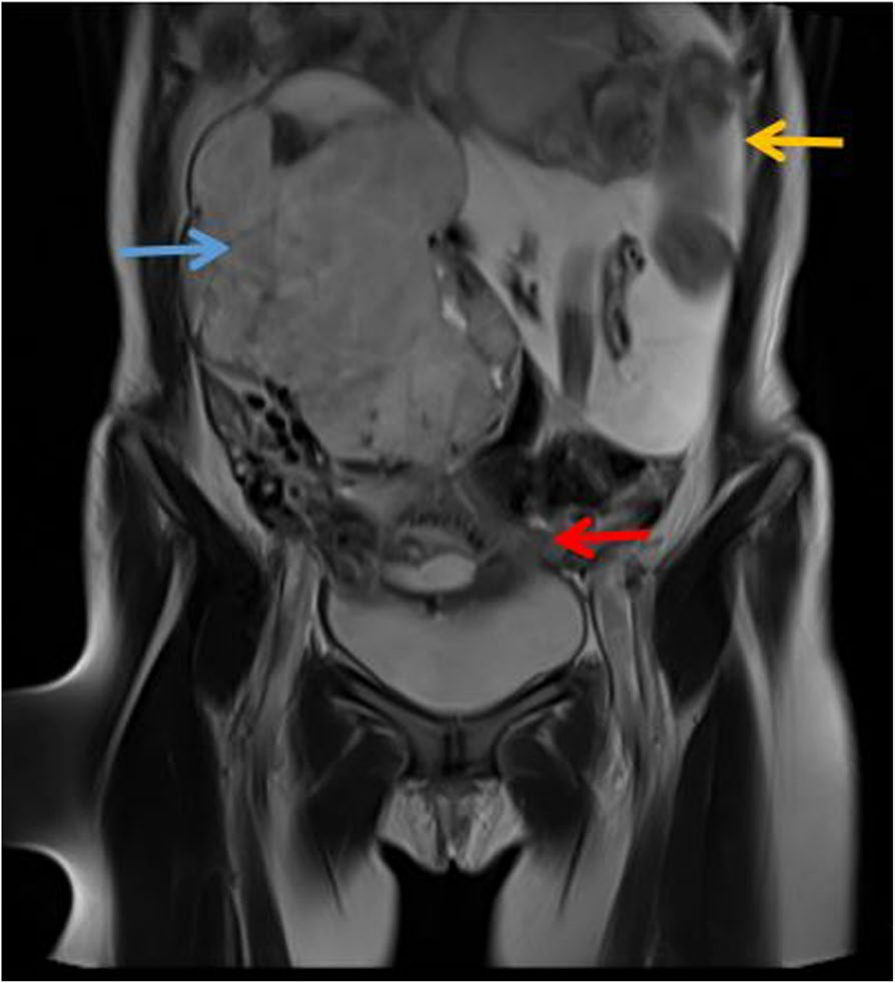
MRI showed a live fetus (yellow arrow) in the abdominal cavity, with the placenta (blue arrow) on the right side of the abdominal cavity rather than in the endometrium (red arrow)

**Fig. 2:**
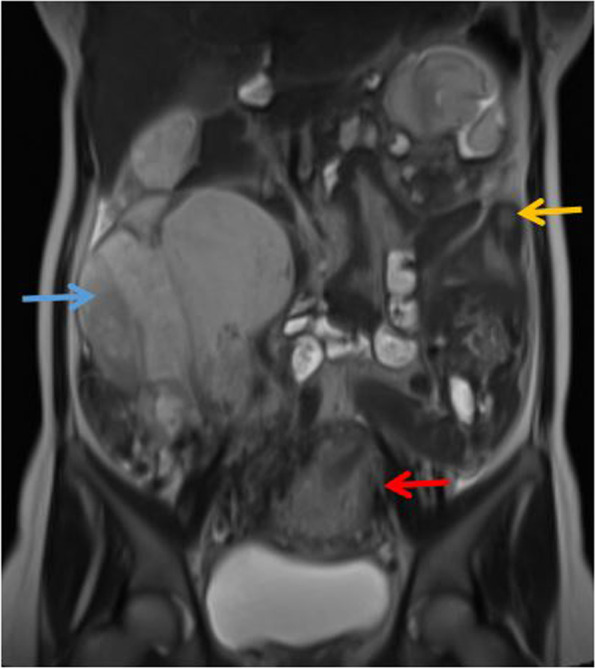
MRI showed an abdominal pregnancy, Placenta (blue arrow), fetus (yellow arrow), and uterus (red arrow)

In September 2021, during the 29th week of her pregnancy, the patient experienced intermittent abdominal pain for 3 h. Magnetic resonance imaging (MRI) (Figs. 1 and 2) revealed an abdominal pregnancy and rupture of the fetal membranes. We performed an emergency laparotomy and found part of the omentum adherent to the anterior abdominal wall and a live fetus of the same gestational age on the left side of the abdominal cavity.

**Fig. 3:**
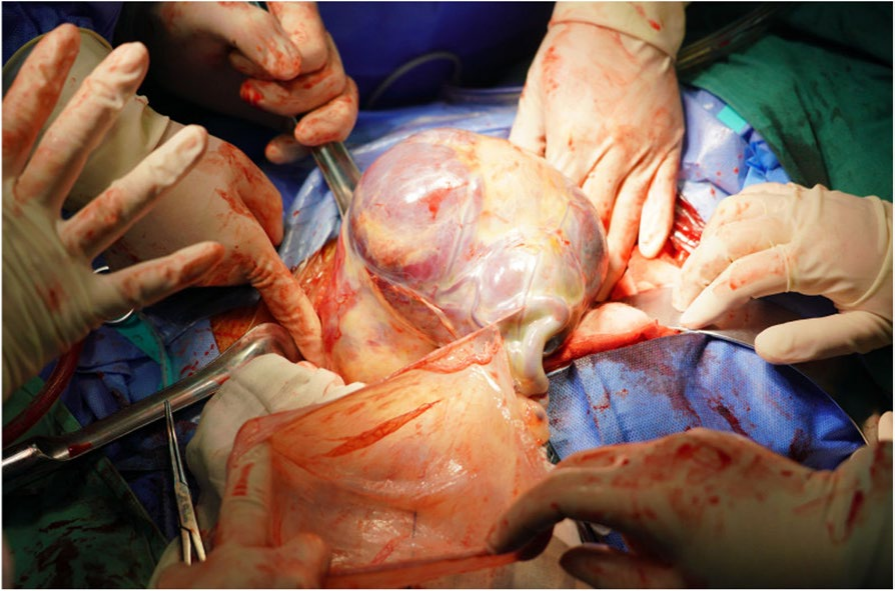
A placentectomy was performed

During the surgery, we performed procedures such as transurethral ureteroscopy, double J-stent placement, transabdominal fetal removal, placentectomy (Fig. 3), right uterine cornual repair, and pelvic adhesiolysis. We found that the amniotic membrane had ruptured, but there were no clear ascites or amniotic fluid in the abdominal and pelvic cavities. In the right pelvic cavity, an amniotic sac covered the placenta, which was attached to the right uterine horn. The placental implantation site was approximately 2 cm × 3 cm, the placental size was approximately 15 cm × 10 cm, and the fetal membrane was wrapped around it. The uterus was enlarged, equivalent to the size of a 60-day gestation, soft, and showed no abnormalities in the appearance of the bilateral appendages. The right fallopian tube and the ovarian ligament were approximately 1 cm from the implantation site. We separated the adhesions between the omentum and the anterior abdominal wall, carefully explored the pelvic and abdominal cavities, and found the placenta implanted in the right uterine horn with no evidence of pelvic, abdominal organ, or large vessel involvement.

We delivered a live male infant in the left sacro-anterior position (LSA) weighing 1200 g, with a body length of 38 cm and a head circumference of 27 cm. The APGAR scores at 1 min and 5 min after birth were 10 and 10, respectively. The placenta weighed approximately 500 g.

The placenta was attached to the right uterine horn, and the left uterine horn was normal. The fetus and placenta entered the abdominal cavity by rupturing the right uterine horn. The placenta was attached to the right uterine horn, sealing the rupture. The surgeon repaired the right uterine horn after delivering the placenta.

The patient had an uneventful recovery and was discharged eight days after surgery. The discharge diagnosis was rudimentary uterine horn pregnancy and secondary abdominal pregnancy. Immediately after birth, the newborn was transferred to the Neonatal Intensive Care Unit (NICU) for further management. He was diagnosed with bronchopulmonary dysplasia, neonatal respiratory distress syndrome, intrauterine infectious pneumonia, premature encephalopathy, jaundice of premature infants, anemia of premature infants (moderate), sclerema neonatorum (mild), hyperthermia, and fetal growth restriction. He also had hypoalbuminemia, hyperkalemia, and vitamin D deficiency. Eventually, he was discharged from the hospital after 41 days of NICU management. A follow-up was scheduled for this patient, but she did not have regular follow-up.

## Discussion

This report describes the manifestation, diagnosis, and treatment of a rare clinical condition. The patient’s ultrasound examination at the local hospital suggested a bicornuate uterus. However, during surgery, we found that the patient had a unicornuate uterus instead of a bicornuate uterus, and this rare rudimentary uterine horn pregnancy ended up as a secondary abdominal pregnancy. This highlights the importance of using surgical findings for making a final diagnosis in such cases.

Surgical management of ectopic pregnancy is required when a patient presents with hemodynamic instability, symptoms of an ongoing ruptured ectopic mass (such as pelvic pain), or signs of intraperitoneal bleeding [7]. The terms ‘uterine horn’, ‘bicornuate uterus’ and ‘unicornuate uterus’ are often confused. The uterine horn is a normal part of the female internal genitalia that connects the fallopian tubes to the uterus. A bicornuate uterus describes an irregularly shaped uterus with two cavities of varying dimensions, while a unicornuate uterus has only one fallopian tube and an abnormally shaped uterine cavity. Both bicornuate and unicornuate uteri are congenital uterine anomalies that are relatively rare and can lead to pregnancy complications.

Ectopic pregnancy is a serious acute abdominal condition that occurs in 1 to 2% of all pregnancies. Uterine horn pregnancy is an exceedingly rare condition that arises from the implantation and subsequent growth of a fertilized ovum within a rudimentary horn of the uterus. The muscular wall of the congenitally malformed uterus is often dysplastic and cannot support the growth and development of the fetus. Complete or incomplete rupture of the myometrium usually occurs between 14 and 20 weeks of gestational age. After uterine rupture, the fetus is extruded into the abdominal cavity, and the placenta adheres to the pelvic peritoneum and adjacent organs, as occurred in this case. In an abdominal pregnancy, abnormal placentation and insufficient blood supply make it difficult for the fetus to survive to full term. Fortunately, the placenta was attached to the right uterine horn with adequate blood supply, allowing the fetus to survive until 28 weeks of gestational age.

Diagnosing uterine horn pregnancy is difficult, particularly in the early stages, when typical symptoms are absent or may not appear until the second trimester. Therefore, we should focus on high-risk factors for ectopic pregnancy, such as uterine malformation, assisted reproduction, endometriosis, a history of pelvic inflammatory disease and conservative fallopian tube treatment [8]. Currently, Studdiford’s criteria are often used clinically to diagnose abdominal pregnancy. A primary abdominal pregnancy occurs when the embryo embeds in the normal fallopian tubes and ovaries without uteroplacental fistula and attaches completely to the peritoneal surface in the early stages of pregnancy [5]. Abdominal pregnancies usually present with abdominal or gastrointestinal symptoms during pregnancy [9], but their diagnosis is often challenging. Although prenatal follow-up and ultrasound scans are commonplace, many cases are still missed, and 40–50% of cases are diagnosed during surgery [10]. A careful abdominal examination often reveals a uterus with barely recognizable normal contour but easily palpable fetal limbs, abnormal fetal position, abnormally clear fetal heart sounds, and a loud placental murmur. If the B-mode ultrasound diagnosis is inconclusive or highly suggestive of abdominal pregnancy, MRI should be performed when the patient’s condition is stable [11]. In poor economic areas where MRI examination is unavailable, or the professional skills of B mode ultrasound doctors are limited, clinicians must always have a high index of suspicion in patients with fetal motion pain to enhance the safety of both fetus and mother [12].

The incidence of abdominal pregnancy among all ectopic pregnancies is less than 1%, but the mortality rate is much higher than that of tubal pregnancy [13, 14]. Therefore, surgery is recommended immediately after the diagnosis of abdominal pregnancy. However, in rare cases, if maternal and fetal monitoring is normal, pregnancy may continue into the fetal survival period [15].

As the placenta can attach to the uterine wall, intestine, mesentery, liver, spleen, bladder, or ligaments, women with abdominal pregnancies are at high risk of bleeding during surgery. In 2018, Marcelin et al*.* reported an advanced abdominal pregnancy treated with embolization of the placenta after removal of the fetus by laparotomy. Following reduced blood supply, the placenta was resected four weeks later, with reduced blood loss and risk of organ injury [16]. Preoperative evaluation and preparation are critical, including preoperative vascular embolization, uterine artery embolization, ureteral stenting, intestinal preparation, adequate blood preparation, and involvement of a multidisciplinary rescue team. The management of the placenta should be considered carefully. Removing the placenta after the delivery of the fetus during surgery would depend on the placental attachment site and whether the fetus is alive or dead.

We reviewed the literature to identify other case reports of abdominal ectopic pregnancies. We searched PubMed and Google Scholar using the search terms “unicornuate uterus,” “bicornuate uterus,” “ectopic pregnancy,” “abdominal ectopic pregnancy,” and “heterotopic pregnancy”. The reported cases and data are summarized in Table 1. The literature described different laparoscopic approaches for rudimentary horn pregnancies, most of which involved pregnancies in the first or second trimester. With the help of ultrasound, the diagnosis was missed in only one case. The preoperative diagnosis in these cases included isthmic, ectopic, tubal ectopic, cornual, abdominal, rudimentary horn pregnancies, and even acute abdomen. The pregnancy was in the left horn in 4 of the 11 cases, and one case involved a ruptured abdominal pregnancy.

**Table 1:** The reported cases and available data

Author	Age of Patient	Obstetric History	Gestational Age in Weeks	Symptoms	Diagnostic Tool	Preoperative Diagnosis	Treatment	Rupture	RHP side	Live fetus or not	Other Findings	pregnancy outcome
Thang NM et al. (2021) [17]	32	G1	8	mild abdominal pain	Transvaginal Doppler Ultrasound/CT	ectopic pregnancy	laparoscopic surgery twice	no	right	no	placenta implanted on the anterior wall of the rectum	termination of pregnancy
Ku CW et al. (2022) [8]	30		8.9	sharp colicky abdominal pain/nausea/non-bilious vomiting/diarrhea	hysterosalpingo-foam sonography	left ectopic tubal pregnancy	four-port laparoscopy	no	left	IVF, double ET, one abdominal heterotopic pregnancy, one viable IUP		termination of pregnancy
Hailu FG et al. (2017) [9]	26	G4	37	vomiting, epigastric pain, headache, and blurring of vision	ultrasonography	advanced abdominal pregnancy	Emergency cesarean	no	right	yes		Live birth
Sib SR et al. (2018) [10]	22	G4	not reported	bowel sub-obstruction and intrauterine fetal death	no ultrasound or blood tests	abdominal pregnancy	laparotomy	yes	left	no		not reported
Paluku et al. (2020) [12]	25	not reported	33	severe abdominal pain during fetal movements	Ultrasound	acute abdomen	Emergency laparotomy	no		yes	placenta implanted on the greater omentum and the small bowel mesentery	live birth
Dicker et al. (1998) [18]	31	G1	8	right lower quadrant pain	Ultrasound	cornual pregnancy	LS-RHE; bipolar forceps	no	right	no		termination of pregnancy
Yahata et al. (1998) [19]	22	G1	7	none	Ultrasound	RHP/isthmic/cornual pregnancy	LS-RHE; stapling device	no	right	no	right uterine artery attached to the left unicornuate uterine horn	termination of pregnancy
Edelman et al. (2003) [20]	24	G2P0010	7	failed surgical and medical abortion	US, followed by MRI	rudimentary horn pregnancy	MTX followed by morcellator	no	right	no		termination of pregnancy
Chakravati et al. (2003) [21]	23	not reported	9	vaginal bleeding		ectopic pregnancy	LS-RHE	no	left	yes		termination of pregnancy
Sonmezer et al. (2005) [22]	28	G1	6	none	Ultrasound		LS-RHE; bipolar forceps; endobag	no	right	no		termination of pregnancy
Henriet et al. (2008) [23]	28	G2P0010	7	none	Ultrasound	rudimentary horn pregnancy	LS-RHE	no	left	no	with placenta percreta and endometriosis	termination of pregnancy

## Conclusions

Abdominal pregnancy resulting from uterine horn pregnancy is extremely rare. Early diagnosis is crucial for patients at high-risk, including those with uterine malformations, a history of ectopic pregnancy or pelvic inflammatory disease, and those who underwent assisted reproduction. A high index of suspicion and appropriate investigations are necessary to identify this condition. Secondary abdominal pregnancy arises when the pregnancy is located outside the uterus, leading to potentially adverse maternal and fetal issues. Failure to diagnose a uterine horn pregnancy promptly could cause cornual rupture and internal hemorrhage. This case report emphasizes the importance of early detection and management of abdominal pregnancy.

## Data Availability

The datasets used and/or analyzed in this study are available from the corresponding author upon reasonable request.

## Abbreviations

MRI: Magnetic Resonance Imaging
LSA: Left Sacro-Anterior
HCG: Human Chorionic Gonadotropin
RHP: Rudimentary Horn Pregnancy
LS-RHE: Laparoscopic Rudimentary Horn Excision
